# Early Diagnosis of CNS Virus Infections from Neurological Autoimmune Diseases: A Cross-Sectional Study from China in ER Setting

**DOI:** 10.3390/brainsci14090888

**Published:** 2024-08-30

**Authors:** Daiquan Gao, Xue Lv, Zuoyao Shen, Huicong Wang, Wenfeng Zhao, Huang Wang, Xiukun Jin, Liuchen Tan, Lu Yin, Junhui Wang, Weihua Yue, Hongxing Wang

**Affiliations:** 1 Division of Neuropsychiatry and Psychosomatics, Department of Neurology, Xuanwu Hospital of Capital Medical University, National Center for Neurological Disorders, National Clinical Research Center for Geriatric Diseases, Beijing Psychosomatic Disease Consultation Center, Capital Medical University, Beijing 100053, China; gaodaiquan@xwhosp.org (D.G.); 15652523402@163.com (H.W.); fengfeng_zw@ccmu.edu.cn (W.Z.); wanghuang1118@163.com (H.W.); jinxiukun1997@163.com (X.J.); tanliucen233@163.com (L.T.); 2 The First Affiliated Hospital of Xinxiang Medical College, Xinxiang 453100, China; xue_l2022@163.com (X.L.); 18800156030@163.com (Z.S.); 3 NHC Key Laboratory of Mental Health (Peking University), National Clinical Research Center for Mental Disorders (Peking University Sixth Hospital), Beijing 100191, China; 4 Medical Research & Biometrics Centre, Fuwai Hospital, National Centre for Cardiovascular Diseases, Peking Union Medical College, Chinese Academy of Medical Sciences, Beijing 102300, China; yinlu@fuwai.com; 5 Lunenfeld-Tanenbaum Research Institute, Mount Sinai Hospital, Toronto, ON M5G 1X5, Canada; 6 Sunsimiao Hospital, Beijing University of Chinese Medicine, Tongchuan 727000, China; 7 Beijing Institute of Brain Disorders, Beijing 100069, China

**Keywords:** central nervous system, virus infections, neurological autoimmune diseases, differentiation

## Abstract

It is challenging to differentiate between central nervous system (CNS) virus infections and neurological autoimmune diseases in the emergency department. Considering their different pathogenesis, we assume they differ in neuropsychiatric symptoms and laboratory results. A total of 80 patients were included in this study, 50 with CNS virus infections and 30 with CNS autoimmune diseases, confirmed by a polymerase chain reaction (PCR) of cerebrospinal fluid (CSF). A binary logistic regression model and receiver operating characteristic (ROC) curve were employed to examine the discrimination between the two types of diseases based on neuropsychiatric symptoms and laboratory results. Compared to patients with neurological autoimmune diseases, patients with CNS virus infections had a higher incidence of abnormal behavior (*p* = 0.026) and abnormal sensation/thought (*p* = 0.029); higher total (*p* = 0.005), direct (*p* = 0.004), and indirect bilirubin (*p* = 0.004); and increased CSF cell (*p* = 0.01) and CSF white cell counts (*p* = 0.01). Patients with disturbance of consciousness and abnormal sensation/thought were 7.79-fold and 5.07-fold more likely to be diagnosed with CNS virus infections (*OR* = 7.79, *p* = 0.008; *OR* = 5.07, *p* = 0.032). Each unit increase in blood indirect bilirubin concentration and CSF white cell counts increased the risk of developing CNS virus infections by 1.25-fold and 1.01-fold (*OR* = 1.25, *p* = 0.016; *OR* = 1.01, *p* = 0.011). ROC analysis showed that the area under the curve was 88.0% (*p* < 0.001). Our study found that patients with CNS viral infections tend to have higher blood indirect bilirubin concentration, CSF leukocyte count, frequency of disorders of consciousness, and abnormal sensation and thought, which may help differentiate them from those with neurological autoimmune diseases.

## 1. Introduction

Neuropsychiatric symptoms in an emergency setting, which mainly include abnormal behavior, negative and positive symptoms, disturbance of consciousness, memory deficits, etc. [1], can manifest in both central nervous system (CNS) virus infections and neurological autoimmune diseases, which are diagnosed based on the presence or absence of a clear etiology [1,2,3,4]. Clinically, only based on the initial neuropsychiatric symptoms, without any other obvious physical signs, it is hard to differentiate CNS infections, such as Herpes simplex virus type 1 (HSV-1) encephalitis [5], from neurological autoimmune diseases, such as autoimmune encephalitis [6,7]. In some cases, patients who had neuropsychiatric symptoms may even be treated with only antipsychotics without controlling the actual etiology, which would aggravate the symptoms of patients and even cause adverse outcomes [8]. Due to their different causes and clinical interventions, early, accurate identification is key for those with shared neuropsychiatric symptoms [2,4].

Studies have identified that infections, such as HSV [9], and autoimmune diseases, such as anti-NMDA receptor encephalitis [4], play indispensable roles in inducing neuropsychiatric symptoms in different ways [10,11,12,13,14]. Infections can affect the permeability of the blood–brain barrier (BBB) through a series of inflammatory reactions and further trigger neuropsychiatric symptoms [15], which is consistent with the findings of studies exploring biomarkers in the blood and cerebrospinal fluid associated with mental disorders [16]. These studies have found changes in antibodies, albumin ratios, total protein, immunoglobulins, and cytokines, suggesting that BBB leakage or dysfunction may exist in patients with psychiatric disorders and induce neuropsychiatric symptoms [17]. Unlike infections, autoimmune diseases may cause brain dysfunction through autoantibodies [1,2]. Antibodies against neuronal cell surface proteins, ion channels, or receptors have been found in patients with autoimmune encephalitis. The effects of brain-reactive antibodies have also been confirmed by experimental animal studies, which have found that brain-reactive antibodies can induce psychotic-like symptoms [18]. The risk factors of autoimmune diseases include genetic susceptibility and infections [19]. Therefore, these existing results suggest that the combination of blood biomarkers, cerebrospinal fluid (CSF), and mental symptoms helps establish early clinical composite indicators that can distinguish these two types of diseases in the early stage.

However, few studies on the changes in CSF in Chinese patients with neuropsychiatric symptoms have been reported. We hypothesized that patients with CNS viral infections would exhibit different incidences of neuropsychiatric symptoms and variations in internal molecular biomarkers compared to those with neurological autoimmune diseases. Therefore, we utilized the data of neuropsychiatric symptoms and laboratory inspection results of patients with the two types of conditions to explore the differences in the incidence of neuropsychiatric symptoms and internal molecular biomarkers between these two groups. We expected to differentiate the internal molecular biomarkers of CNS virus infections from those of neurological autoimmune diseases.

## 2. Methods

### 2.1. Participants and Data Acquisition

Patients included in this study were recruited through the emergency neurology department at Capital Medical University. Recruitment and data collection were conducted between 2020 and 2021. Patients diagnosed with CNS virus infections and CNS autoimmune diseases were included. Patients with a history of epilepsy or substance dependence were excluded. Laboratory tests were conducted on participants, including blood chemistry analysis (uric acid, creatinine, lactate dehydrogenase, creatine kinase, urea, total bile acids, indirect bilirubin, direct bilirubin, total bilirubin, low-density lipoproteins [LDLs], high-density lipoproteins [HDLs], triglycerides, and albumin) and CSF analysis (cell counts for both white and red blood cells, separate white cell counts, glucose, chloride, protein, lactic acid, IgG, IgM, IgA, and the IgG intrathecal synthesis rate).

The data collection was fully anonymized and was approved by the ethics committee of Xuanwu Hospital of Capital Medical University.

### 2.2. Subgroup Definition

Diseases included CNS virus infections and neurological autoimmune diseases, diagnosed by the National Center for Neurological Disorders of China and confirmed with PCR for viral DNA or RNA or with a Cell-Based Assay for specific autoantibodies in CSF (for details of technologies, see Supplementary Materials). The detailed information of the patients included in this study is provided in Table S1. Neuropsychiatric symptoms were evaluated by clinicians and divided into the following categories based on psychopathology: abnormal behavior, impaired memory, abnormal sensation and thought (including illusions, delusions, and aphasia), and disturbance of consciousness [1].

### 2.3. Statistical Analyses

Statistical analyses were conducted in IBM SPSS Statistics 25 and RStudio. Categorical variables were compared using chi-square tests between CNS virus infections and CNS autoimmune diseases, and continuous variables were compared using Wilcoxon rank-sum tests. Associations among diagnosis, neuropsychiatric symptoms, and laboratory inspection results were analyzed using binary logistic regression. Sex and age were included as covariates owing to their putative effects. A *p*-value < 0.05 was considered significant. The significant area under the curve (AUC) was used as an effective model to the discrimination of CNS virus infections from neurological autoimmune diseases. An area under the curve of at least 0.7 was considered satisfactory, and the null hypothesis value was set at 0.5.

## 3. Results

### 3.1. Demographics and Incidence of Neuropsychiatric Symptoms

A total of 80 patients were included in this study (Table 1), of whom 50 had CNS virus infections, and 30 had CNS autoimmune diseases. There were no significant differences in age (*p* = 0.988) between the two groups and the age composition ratio of the patients with different diseases (*p* = 0.602) (Figure S1). There was a slightly higher proportion of males in all patients, with no significant group difference (*p* = 0.684).

Abnormal behavior is more frequent in patients with CNS virus infections (22.0%) than in those with neurological autoimmune diseases (3.3%), with a significant group difference (*p* = 0.026); abnormal sensation and thought is more frequent in patients with CNS virus infections (44.0%) than with neurological autoimmune diseases (20.0%), with a significant group difference (*p* = 0.029). Besides these, the differences in the other incidences of neuropsychiatric symptoms in the two groups failed to reach statistical significance.

### 3.2. Association between Laboratory Inspection Results and Diseases

For blood biochemistry, the medians of the concentrations of total bilirubin (*p* = 0.005), direct bilirubin (*p* = 0.004), and indirect bilirubin (*p* = 0.004) were higher in patients with CNS virus infections than in those with neurological autoimmune diseases. However, the differences in other blood biochemistry items between the two types of conditions did not reach statistical significance (Table 2).

For the CSF test results, the medians of the CSF cell counts (*p* = 0.01) and CSF white cell counts (*p* = 0.01) were higher in patients with CNS virus infections compared to patients with neurological autoimmune diseases. Apart from these, the differences in other CSF tests between the two types of conditions did not reach statistical significance (Table 3). Further mediation analysis revealed that CSF white cells did not mediate the effect of CSF cells on disease with the 97.5% confidence interval of Za×Zb containing zero (97.5% CI, −1.40 × 10^−5^ to 3.87 × 10^−4^).

### 3.3. Multivariable Analysis

**Figure 1:**
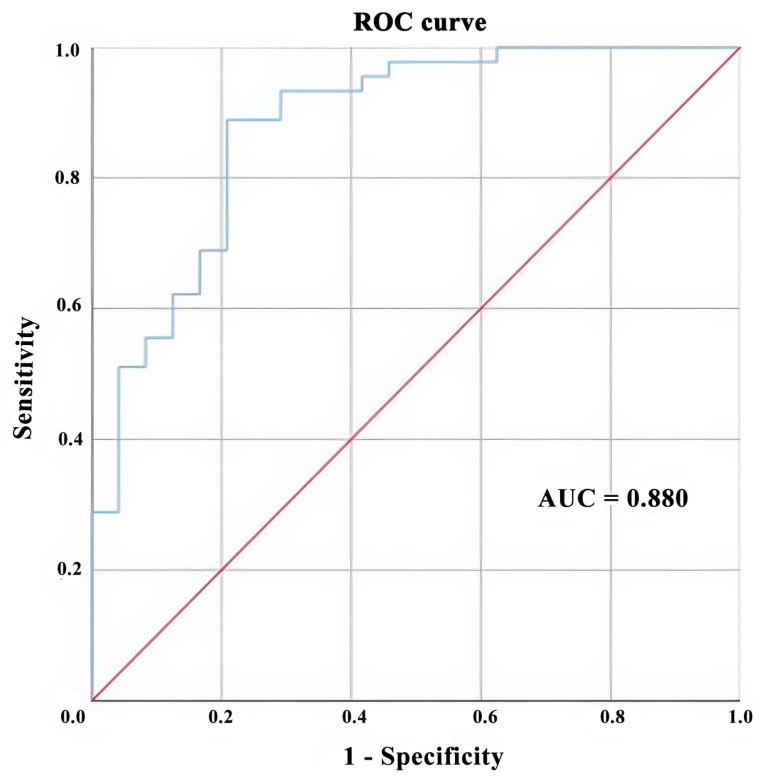
The ROC curve of the model to differentiate CNS virus infections with neurological autoimmune diseases based on abnormal sensation and thought, disturbance of consciousness, blood direct bilirubin concentration, and CSF white cell counts. The red line represents the performance of a random classifier with an AUC of 0.5; the blue line shows the ROC curve of our model. AUC, the area under the ROC curve; ROC curve, receiver operating characteristic curve.

Sex, age, CSF cell counts, CSF white cell counts, blood direct bilirubin concentration, blood indirect bilirubin concentration, and neuropsychiatric symptoms (abnormal behavior, impaired memory, abnormal sensation and thought, and disturbance of consciousness) were put in the model of binary logistic regression with forward LR. Ultimately, CSF white cell counts, blood indirect bilirubin concentration, abnormal sensation and thought, and disturbance of consciousness were selected to be associated with different diagnoses (Table 4). Patients with disturbance of consciousness were 7.79-fold more likely to be diagnosed with CNS virus infections than those without this symptom (*OR* = 7.79; *p* = 0.008); patients with abnormal sensation and thought were 5.07-fold more likely to be diagnosed with CNS virus infections than those without this symptom (*OR* = 5.07; *p* = 0.032). Each unit increase in blood indirect bilirubin concentration was associated with a 1.25-fold increase in the risk of developing CNS virus infections rather than neurological autoimmune diseases (*OR* = 1.25; *p* = 0.016), and each unit increase in CSF white cell counts was associated with a 1.01-fold increase in the risk of developing CNS virus infections rather than neurological autoimmune diseases (*OR* = 1.01; *p* = 0.011). Subsequently, receiver operating characteristic (ROC) analysis was performed to evaluate the diagnostic performance of the four aforementioned indexes. Figure 1 shows that the area under the curve was 88.0% (AUC = 0.880, SE = 0.045, 95% *CI* = 0.792–0.967, *p* < 0.001), indicating that, based on the disturbance of consciousness, abnormal sensation and thought, CSF white cell counts, and indirect blood bilirubin, the probability of successfully differentiating CNS viral infections from neurological autoimmune diseases could reach 88%.

## 4. Discussion

The main finding of this study is the confirmation that patients with CNS virus infections tend to have higher blood indirect bilirubin concentration and CSF white cell counts and a higher frequency of disturbance of consciousness and abnormal sensation and thought than patients with neurological autoimmune diseases. In addition, the present study demonstrates that the diagnostic accuracy is relatively sufficient for differentiating CNS virus infections with the four aspects mentioned above.

### 4.1. Neuropsychiatric Symptoms

The pathophysiology of neuropsychiatric symptoms has been explored in previous studies [20,21]. Age has previously been shown to be a risk factor for disturbance of consciousness, which is more likely to occur with increasing age [22,23]. However, further comparison showed that the difference in the age composition ratio between the two types of diseases included in this study failed to reach statistical significance, which supports that the incidence of disturbance of consciousness is significantly different in the two conditions.

Studies have explored the associations between CNS virus infections and mental disorders. HSV-1 infections have been identified to affect the permeability of the blood–brain barrier and gray matter volume, which are the foundation of developing mental disorders; cognitive impairment has also been proven to be associated with herpes virus infections [24,25,26,27].

The mechanisms by which brain-reactive antibodies trigger neuropsychiatric symptoms have been increasingly explored. Previous studies showed that brain-reactive antibodies may target NMDARs in the brain, leading to their removal from the synapse and causing mental symptoms, which is in line with the glutamatergic model of schizophrenia [28]. However, the finding that brain-reactive antibodies can also be found in healthy people afford much food for thought. Recent reports revealed that the seroprevalence, immunoglobulin class, or titers of humoral autoimmunity against brain antigens do not predict disease [29]. That is, the effects of brain-reactive antibodies need more exploration.

### 4.2. Internal Molecular Biomarkers

The association of patients with CNS virus infections with increased CSF white cell counts is in accordance with previous studies. Increased CSF white cell counts are a signal of an inflammatory response in the central nervous system, and chemoattractant cytokines are thought to play a crucial role in controlling white cells in the subarachnoid space [30,31]. Therefore, increased CSF white cell counts further verified the effects of CNS virus infections on the blood–brain barrier.

Blood indirect bilirubin has been identified as an antioxidant that is crucial for neutralizing reactive oxygen species (ROS) and is believed to mediate the trans-endothelial migration of monocytes, thereby affecting the permeability of the blood–brain barrier [32]. Blood indirect bilirubin is also found to have potential immunomodulatory properties. Our finding that the concentration of blood indirect bilirubin in patients with CNS virus infections is slightly higher than that of patients with neurological autoimmune diseases is in line with previous studies, which have found that the concentration of blood indirect bilirubin decreases in patients with neurological autoimmune diseases like Guillain–Barré syndrome and multiple sclerosis [33,34]. A potential reason for this has been discussed in earlier studies, suggesting that autoimmune alterations in patients with neurological autoimmune diseases may lead to changes in liver function and oxidative stress responses, resulting in lower blood indirect bilirubin levels [33]. In contrast, viral infections can lead to liver involvement and systemic inflammation, which may help explain why blood indirect bilirubin levels are higher in patients with CNS viral infections compared to those with neurological autoimmune diseases [35]. However, absolute blood indirect bilirubin concentrations in the two disease groups in this study differ widely from previous studies. These differences may be attributable to the severity of diseases and sample size, and further studies are needed.

### 4.3. Strengths and Limitations

This study has several strengths. To our knowledge, this was the first study to investigate whether the differences in the internal molecular biomarkers and the incidence of neuropsychiatric symptoms can help to distinguish Chinese patients with CNS virus infections from those with neurological autoimmune diseases early and precisely. This study included CSF biochemistry results, which can reveal the changes in the brain more directly. Neuropsychiatric symptoms were classified into specific categories rather than sweeping generalizations, which will not only help clinicians to diagnose these diseases more efficiently but also further research into the mechanisms of different neuropsychiatric symptoms.

The limitations of this study should also be noted. In our cross-sectional study, the status of CNS virus infections of patients with neurological autoimmune diseases was not learned. For example, one study showed that the prognosis of patients with autoimmune encephalitis after herpes simplex encephalitis was substantially worse than that reported in patients with classical anti-NMDAR encephalitis [9]. Additionally, this study lacked comparisons with healthy controls and patients with functional mental disorders. A small sample size and lack of quantitative scores for mental symptoms may also lead to bias in the results. Additionally, we focused on a limited set of laboratory examination indicators in this study. Future research could incorporate a broader range of indicators to enhance the early differentiation of these two types of diseases and to further elucidate their pathogenesis, thereby providing guidance for timely and effective treatment.

## 5. Conclusions

In conclusion, we have identified that the disturbance of consciousness, abnormal sensation and thought, CSF white cell counts, and indirect blood bilirubin can jointly contribute to differentiating CNS virus infections from neurological autoimmune diseases, which may help make appropriate decisions about further treatment and controlling disease progression at an earlier stage.

## Figures and Tables

**Table 1:** Demographics and incidences of neuropsychiatric symptoms of studied patients.

	CNS Virus Infections (*n* = 50)	Neurological Autoimmune Diseases (*n* = 30)	*p*-Value
Sex (male/female)	29/21	16/14	0.684
Age (years), Mean (SD)	48.7 (17.6)	48.6 (16.9)	0.988
Abnormal behavior (without/with)	39/11	29/1	0.026 *
Impaired memory (without/with)	48/2	27/3	0.358
Disturbance of consciousness (without/with)	30/20	24/6	0.064
Abnormal sensation and thought (without/with)	28/22	24/6	0.029 *

The *p*-value was obtained by a chi-square test and *t*-test. CNS, central nervous system; SD, standard deviation. *p*-value with * indicates *p* < 0.05.

**Table 2:** Blood biochemistry result details of patients with different diagnosis.

	CNS Virus Infections (*n* = 50)		Neurological Autoimmune Diseases (*n* = 30)		*p*-Value
	Median	[Q1, Q3]	Median	[Q1, Q3]	
Uric acid (μmol/L)	269	[178, 391]	252	[220, 331]	0.833
Creatinine (μmol/L)	61	[46.3, 71.8]	52	[43.0, 65.8]	0.278
Lactate dehydrogenase (IU/L)	227	[189, 301]	189	[168, 246]	0.063
Creatine kinase (IU/L)	122	[60.0, 457]	91	[48.0, 140]	0.074
Urea (mmol/L)	4.7	[3.47, 5.68]	4.7	[4.32, 6.50]	0.611
Total bile acids (μmol/L)	2.5	[1.73, 4.13]	4.8	[2.40, 6.20]	0.060
Indirect bilirubin (μmol/L)	9.07	[7.24, 13.1]	6.19	[5.23, 9.24]	0.005 *
Direct bilirubin (μmol/L)	4.56	[3.49, 6.28]	2.86	[1.98, 4.87]	0.004 *
Total bilirubin (μmol/L)	14.3	[10.8, 19.5]	9.32	[6.73, 13.4]	0.004 *
LDL (mmol/L)	2.42	[1.91, 3.02]	2.56	[2.27, 3.06]	0.447
HDL (mmol/L)	0.975	[0.793, 1.43]	1.21	[0.905, 1.44]	0.278
Triglycerides (mmol/L)	0.97	[0.668, 1.27]	0.92	[0.705, 1.36]	0.656
Albumin (g/L)	40.6	[36.3, 43.3]	41.4	[38.2, 43.6]	0.379

The *p*-value was obtained by a rank-sum test. CNS, central nervous system; HDL, high-density lipoprotein; LDL, low-density lipoprotein. *p*-value with * indicates *p* < 0.05.

**Table 3:** CSF test result details of patients with different diagnosis.

	CNS Virus Infections (*n* = 50)		Neurological Autoimmune Diseases (*n* = 30)		*p*-Value
	Median	[Q1, Q3]	Median	[Q1, Q3]	
CSF cell counts (×10^6^/L)	263.00	[40.0, 1000]	11.50	[2.00, 291]	0.01 *
CSF white cell counts (×10^6^/L)	16.50	[2.75, 140]	2.00	[1.00, 9.00]	0.01 *
CSF glucose (mg/dL)	61.40	[55.3, 73.4]	66.80	[57.8, 75.1]	0.53
CSF chloride (mmol/L)	125.00	[121, 128]	126.00	[123, 130]	0.12
CSF protein (mg/dL)	42.80	[25.4, 89.9]	44.60	[29.7, 54.5]	0.77
CSF lactic acid (mmol/L)	1.95	[1.63, 2.68]	1.80	[1.60, 2.00]	0.12
CSF IgG (mg/dL)	5.46	[3.04, 10.3]	4.61	[2.44, 7.04]	0.55
CSF IgM (mg/dL)	0.21	[0.04, 0.45]	0.09	[0.06, 0.17]	0.14
CSF IgA (mg/dL)	0.78	[0.37, 1.46]	0.46	[0.33, 1.31]	0.40
IgG intrathecal synthesis rate	3.18	[1.52, 9.09]	6.29	[2.35, 9.22]	0.54

The *p*-value was obtained by a rank-sum test. CNS, central nervous system; CSF, cerebrospinal fluid. *p*-value with * indicates *p* < 0.05.

**Table 4:** Details of model for differentiating CNS virus infections with neurological autoimmune diseases.

	Degree of Freedom	*p*-Value	*OR*
Disturbance of consciousness	1	0.008 *	7.79
Abnormal sensation and thought	1	0.032 *	5.07
Blood indirect bilirubin	1	0.016 *	1.25
CSF white cell counts	1	0.011 *	1.01

The *p*-value was obtained by binary logistic regression. CSF, cerebrospinal fluid; *OR*, odds risk. *p*-value with * indicates *p* < 0.05.

## Data Availability

The data that support the findings of this study are available on request from the corresponding author. The data are not publicly available due to privacy or ethical restrictions.

## Supplementary Materials

The following supporting information can be downloaded at https://www.mdpi.com/article/10.3390/brainsci14090888/s1, Figure S1: Age composition ratio of different diseases included in this study; Table S1: Diagnosis codes based on the WHO International Classification of Diseases (ICD) codes from the ICD-10 periods.
