# Supplementary material for: Early Diagnosis of CNS Virus Infections from Neurological Autoimmune Diseases: A Cross-Sectional Study from China in ER Setting
Source: Brain Sci. 2024 Aug 30;14(9):888. doi: 10.3390/brainsci14090888 (PMC11430841; doi:10.3390/brainsci14090888)
Supplement: Supplementary file 1 [file brainsci-14-00888-s001.zip › brainsci-3158882-supplementary.pdf]

Supplementary Information Text for technologies used detect virus or autoantibodies

For the detection of viral pathogens, Polymerase Chain Reaction (PCR) was employed, focusing on pathogens within the TORCH group, including Toxoplasma gondii, Rubella virus, Cytomegalovirus, and Herpes Simplex Virus types 1 and 2. Initially, samples were screened using the Enzyme-Linked Immunosorbent Assay (ELISA) to identify the presence of specific viral antibodies, with reagents obtained from Trinity Biotech. Following the ELISA screening, DNA or RNA was extracted from the patient's cerebrospinal fluid (CSF). Specific primers were then used to amplify viral gene fragments through PCR. The PCR products were subsequently analyzed via agarose gel electrophoresis, with the presence of amplification bands confirmed under UV light, indicating the presence of viral genetic material.

For the detection of autoantibodies, a Cell-Based Assay (CBA) was utilized. In this method, live cells that either naturally express or have been engineered to express the target antigen on their surface or within the cell were employed. The patient's cerebrospinal fluid (CSF) was incubated with these cells, allowing any specific autoantibodies present to bind to their respective antigens. After incubation, the cells were thoroughly washed to remove any unbound antibodies and then incubated with a secondary antibody conjugated to a detectable marker, such as a fluorescent dye. This secondary antibody binds to the human immunoglobulins attached to the cells. The presence and intensity of the fluorescence were measured using flow cytometry or fluorescence microscopy, providing a quantitative or qualitative assessment of autoantibody binding.

Table S1. Diagnosis codes based on the WHO International Classification of Diseases (ICD) codes from the ICD-10 periods.

Disease categories	Specific disease (N)	ICD-10 code
CNS virus infections	Viral meningoencephalitis (17)<br>Viral encephalitis (33)	A86xx02, B00.401-G05.1*
Neurological autoimmune diseases	Acute demyelinating encephalitis (5)<br>Bickerstaff's brainstem encephalitis (15)<br>Autoimmune encephalitis (10)	G37.901, G61.900, G61.000, G61.001

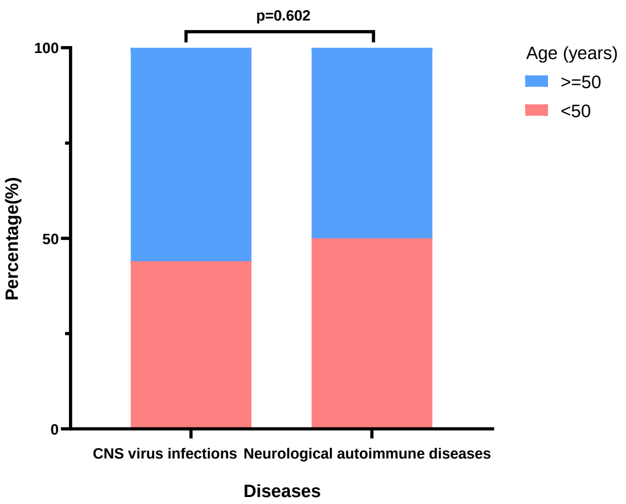

Figure S1. Age composition ratio of different diseases included in this study.
